# Trends in Cartilage Repair Techniques for Chondral Defects in the Hip in Germany: An Epidemiological Analysis from 2006 to 2022

**DOI:** 10.3390/life14101262

**Published:** 2024-10-03

**Authors:** Sebastian Frischholz, Tizian Heinz, Manuel Weißenberger, Sebastian Philipp von Hertzberg-Boelch, Philip Anderson, Martin Lüdemann, Axel Jakuscheit, Maximilian Rudert, Ioannis Stratos

**Affiliations:** Department of Orthopaedic Surgery, University of Wuerzburg, Koenig-Ludwig-Haus, Brettreichstrasse 11, 97074 Wuerzburg, Germany; sebastian.frischholz@uni-wuerzburg.de (S.F.); t-heinz-klh@uni-wuerzburg.de (T.H.); manuel.weissenberger@googlemail.com (M.W.); sebastian.vonhertzberg-boelch@lvr.de (S.P.v.H.-B.); p-anderson.klh@uni-wuerzburg.de (P.A.); m-luedemann.klh@uni-wuerzburg.de (M.L.); a-jakuscheit.klh@uni-wuerzburg.de (A.J.); m-rudert.klh@uni-wuerzburg.de (M.R.)

**Keywords:** cartilage repair techniques, hip, epidemiological analysis, regenerative therapies, age distribution

## Abstract

Cartilage repair techniques for chondral defects in the hip are crucial for treating conditions like femoroacetabular impingement, developmental dysplasia, and osteonecrosis, especially in young patients to delay the progression of osteoarthritis. This study aims to present age and sex distributions and trends in hip-preserving surgeries in Germany from 2006 to 2022, analyzing 116,179 procedures using the German OPS coding system. The procedures were categorized into three groups: debridement, refixation, and regeneration. Arthroscopy was more common than arthrotomy (98,916 vs. 17,263). Males underwent more procedures than females (63,771 vs. 52,408). Debridement had a monomodal age distribution peaking at 43.42 years, while refixation and regeneration exhibited bimodal patterns. Regenerative procedures were primarily performed on younger patients (average 27.73 years). A Joinpoint analysis showed an initial increase in procedures, peaking around 2013, followed by a decline. Arthroscopic procedures peaked at approximately 9000 in 2013, whereas arthrotomies peaked at around 1200 after 2014. The decline in procedures post-2013 may reflect refined surgical indications and a shift towards outpatient settings. These findings underscore the trend towards minimally invasive, scaffold-based treatments, with regenerative techniques showing promising outcomes in younger patients. Future research should focus on prospective comparative studies and cost–benefit analyses to guide clinical decision-making.

## 1. Introduction

Articular cartilage defects impose a tremendous burden on orthopedic patient care as avascular and aneural hyaline cartilage exhibits very limited self-regenerative capacities [1]. Without adequate and effective treatment, chondral defects frequently cause tissue degeneration associated with severe joint pain, loss of function, and eventually the development of osteoarthritis (OA) [2]. Moreover, as life expectancy and medical care is improving, an increasing prevalence of OA must be presumed over the next decades [3]. According to data analyzed during 2013–2015 from the National Health Interview Survey in the U.S., 54.4 million (22.7%) people from the adult population were diagnosed with arthritis, and of these patients, 43.5% suffered limitations in activity levels [4]. In a systemic review and meta-analysis including 31 studies, the pooled worldwide prevalence of hip OA diagnosed using the Kellgren–Lawrence classification (grade ≥ 2) was 8.55%, whereas it was highest in Europe at 12.59% [5].

Indeed, cartilage repair techniques for chondral defects in the hip are pivotal in the treatment of femoroacetabular impingement, developmental dysplasia, osteonecrosis, osteochondrosis dissecans, loose bodies, and trauma [6]. Especially in younger patients, joint preservation is important to delay treatment with total hip arthroplasty [7]. Thereby, depending on the procedure, surgical interventions can be performed by both arthroscopy and arthrotomy. Besides debridement of cartilage defects and microfracture, regenerative techniques have progressively been established clinically.

Debridement of the articular cartilage helps alleviate symptoms such as pain and limited range of motion and is useful in the management of superficial cartilage defects [8,9]. In microfracture, subchondral drilling inducing a clot with mesenchymal stem cells leads to fibrocartilage with inferior biomechanical properties [10,11].

Regenerative techniques for cartilage repair utilizing different cell sources and various biocompatible scaffolds are promising regarding tissue regeneration [12]. Cell-based therapies include autologous chondrocyte implantation (ACI), matrix-induced autologous chondrocyte implantation (MACI) and autogenous matrix-induced chondrogenesis (AMIC). For ACI and MACI, which are performed in a two-stage procedure, chondrocytes are first harvested from a donor site, expanded in vitro and then replanted into the defect site [13]. In a retrospective study comparing arthroscopic debridement versus ACI for the treatment of hip chondral lesions, a better Harris Hip Score (HHS) was reported for patients who underwent ACI when compared to debridement [14]. In MACI, expanded chondrocytes are seeded onto absorbable scaffolds, which are then implanted into the defect site. In case series, MACI has been reported to improve patient-reported outcomes in the treatment of full-thickness cartilage defects in the hip [15,16]. In contrast, AMIC is performed as a one-stage procedure by covering the cartilage defect site with a collagen matrix patch after a microfracture procedure [8]. In a comparative retrospective study, AMIC resulted in improved modified HHS compared to microfracture in acetabular chondral lesions [17]. Overall, several case series and retrospective studies suggest regenerative techniques as promising treatment options.

Despite the high relevance of articular cartilage defects in the hip and numerous treatment options, epidemiological data on cartilage repair techniques in the hip remain scarce. Therefore, the present study aimed to exhibit the age and sex distributions as well as current trends in joint-preserving surgery of the hip in Germany, focusing on the various therapeutic procedures and their development over time.

## 2. Materials and Methods

Within this study, we analyzed a total of 116,179 surgical procedures performed on patients in hospital throughout Germany over a period of 17 years, from 01.01.2006 to 31.12.2022. The register of encoded procedures was compiled by analyzing the datasets labelled “5-801ff_AG_GE_2006-2022” and “5-812ff_AG_GE_2006-2022”. These two documents entitled “Inpatients discharged from the hospital (including deaths and hourly cases) 2006–2022, selected operations 5-801 by age groups, total, number” and “Inpatients discharged from the hospital (including deaths and hourly cases) 2006–2022, selected operations 5-812 by age groups, total, number” were provided by the Federal Statistical Office of Germany. The files can be downloaded from the data repository (see the Data Availability Statement).

Next, the German procedure classification (OPS classification system) was utilized for further data analyses. The surgical procedures for open and arthroscopic joint operations (OPS codes) were 5-801 for open operations and 5-812 for arthroscopic procedures. For further data classification, we examined the OPS codes ending in g, which represent the hip joint.

Three distinct groups were defined as follows: A debridement group, which included surgical interventions like the removal of impaired articular cartilage tissue, chondral debridement, microfracture, and subchondral drilling; a refixation group consisting of procedures such as osteochondral fragment reduction or microfracture combined with fragment refixation; and a regeneration group, which comprised procedures like matrix-induced autologous chondrocyte implantation, transplantation of in vitro tissue culture, transplantation of cartilage, and acellular matrix implantation with opening of the subchondral bone including autogenous matrix-induced chondrogenesis. This allocation was established according to specific OPS codes: debridement (OPS codes 5-801.[0 g, gg, hg] and 5-812.[0 g, eg, fg]), refixation (OPS codes 5-801.[3 g, 4 g] and 5-812.[3 g]), and regeneration (OPS codes 5-801.[kg, cg, bg, ng, pg] and 5-812.[hg, ag, 9 g, gg, mg]) (Table 1). The procedures “removal of osteophytes”, “not specified”, “cartilage graft removal”, and “implantation of metallic cartilage replacement” were not included in the analysis. The gathered data were further categorized into detailed subgroups, enabling an in-depth analysis. This involved sorting the data according to several criteria: age of the patients, which was pooled to 5-year intervals for analysis simplification; gender; the year of operative treatment, spanning from 2006 to 2022; and type of surgical procedure, distinguishing between arthroscopy and arthrotomy.

### 2.1. Data Processing

The datasets “5-801ff_AG_GE_2006-2022” and “5-812ff_AG_GE_2006-2022” obtained were structured as a table containing aggregated demographic information and OPS codes. It encompassed OPS codes starting with 5-801 and 5-812, categorized by age, applying 22 sections (e.g., “under 1”, “1–5”, “5–10”, …, “85–90”, “90–95”, and “over 95”); gender, differentiating “male” and “female” groups; and years of collection, divided into 17 groups from “2006” to “2022”. For data transformation to a list format, the R programming language (courtesy of RStudio PBC; Boston, MA, USA) and the tidyverse package were used. Additionally, Tableau software version 2024.1.2 (developed by Tableau Software; Seattle, WA, USA) was utilized for data reorganization and profound data analysis.

### 2.2. Statistical Analysis

The analysis involved Python scripts aimed at analyzing and visualizing the dataset. The Python code was executed using the Spyder IDE, version 5.5.4 (developed by the Spyder Project Contributors, distributed by Anaconda, Inc., Austin, TX, USA). Gaussian distributions were used to analyze the age peak of each surgical procedure. Initially, the data were imported from an Excel file using the pandas library, and two Gaussian models—a single Gaussian and a sum of two Gaussians—were fitted to the data using non-linear least squares optimization provided by the scipy.optimize module. The models’ fits were evaluated by calculating the Residual Sum of Squares (RSS) and the Akaike Information Criterion (AIC) to determine the best model.

A joinpoint regression analysis was performed to identify points where the linear trend in the data changes significantly, utilizing Joinpoint software version 5.2.0.0 provided by the National Cancer Institute (Rockville, MD, USA). We opted for a constant variance (homoscedasticity) model for the error structure. The error model was selected as it was uncorrelated, assuming no autocorrelation in the data. The parameters calculated included slope, intercept, standard error of the slope, standard error of the intercept, *p*-value, and t-statistic for the slope and intercept.

## 3. Results

During the time period included, i.e., from 2006 to 2022, a total of 116,179 procedures were performed. Overall, arthroscopic procedures were more frequently performed (17,263 arthrotomy vs. 98,916 arthroscopy; ratio = 1:5.7) (Table 2).

Also, significant gender differences were noted. The overall gender ratio (52,408 female vs. 63,771 male; ratio = 1:1.22) showed that male patients were more common. Both in procedures utilizing arthroscopy and arthrotomy, more procedures were performed on men (44,524 female vs. 54,392 male; ratio = 1:1.22 for arthroscopy and 7884 female vs. 9379 male; ratio = 1:1.19 for arthrotomy) (Table 2).

**Figure 1:**
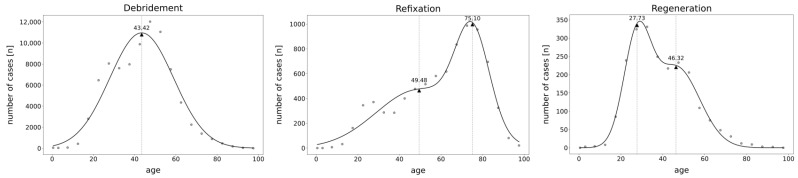
Gaussian distribution analysis of cartilage repair techniques in the hip from 2006 to 2022, categorized by patient age for the regenerative, debridement, and refixation OPS codes. Triangles represent calculated age maxima. Dots represent observed number of cases.

The age distribution showed both mono- and bimodal patterns depending on the procedure (Figure 1). Whereas in the “debridement” group, a monomodal pattern with a calculated peak age of 43.42 years was observed, both the “refixation” and “regeneration” group exhibited bimodal patterns. Thereby, one peak emerged at the age of 45–50 for both procedures (calculated age maximum for “refixation”: 49.48 years, “regeneration”: 46.32 years) (Figure 1). However, the maximum apex was different between the “refixation” and “regeneration” procedures, with patients receiving “refixation” procedures being 75 years old on average (calculated age maximum for “refixation”: 75.10 years). In contrast, “regeneration” patients were nearly 47 years younger averaging 28 years old (calculated age maximum for “regeneration”: 27.73 years) (Figure 1). Thus, regenerative therapies of the hip were primarily conducted in younger patients, as evidenced by the distinctly higher peak in the age distribution curve.

The majority of surgical interventions was categorized as debridement (n = 83,481), whereas refixation (n = 7978) and regeneration (n = 2188) procedures were performed less frequently (Table 3). Chondroplasty and microfracture were predominantly performed in arthroscopic procedures (73,580 arthroscopies vs. 9901 arthrotomies; ratio = 1:0.13) (Table 3). In contrast, refixation surgeries were carried out via arthrotomy more frequently than arthroscopy (1944 arthroscopies vs. 6034 arthrotomies; ratio = 1:3.10). Regarding regenerative procedures, MACI was more frequently performed utilizing arthrotomy rather than arthroscopy (196 arthroscopies vs. 544 arthrotomies; ratio = 1:2.78).

Albeit, AMIC was carried out more often via arthroscopy (846 arthroscopies vs. 325 arthrotomies; ratio = 1:0.38). Interestingly, regenerative surgery was more frequently performed in male than female patients (731 female vs. 1457 male; ratio = 1:1.99) (Table 3).

**Figure 2:**
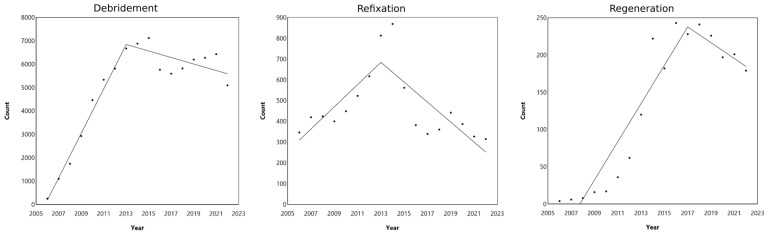
Joinpoint analysis of cartilage repair techniques in the hip from 2006 to 2022, categorized by year for the debridement, refixation, and regenerative OPS codes. Dots represent observed number of cases.

The joinpoint analysis of surgical procedures for “debridement”, “refixation”, and “regeneration” from 2006 to 2022 showed distinct trends in the number of operations over time (Figure 2). For the “debridement” group, the count of procedures increased significantly from 2006, with a peak around 2013, with over 7000 cases.

However, after 2013, the numbers declined. A similar trend was observed for the refixation group, with a steady increase from 2006, reaching an apex around 2013, with over 800 procedures (Figure 2). In contrast, for the “regeneration” group, the number of cases rapidly increased from 2010 until around 2017, with approximately 240 procedures (Figure 2). After that, the number of regenerative surgeries declined. Overall, the joinpoint analysis indicates that after significant increases leading to respective peaks, all included procedures exhibited a decline in frequency.

**Figure 3:**
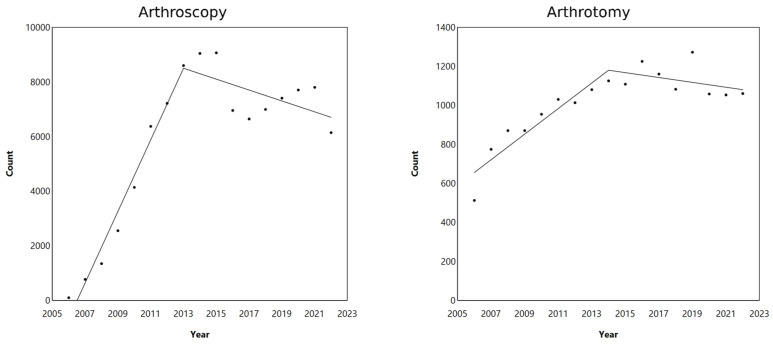
Joinpoint analysis of cartilage repair techniques in the hip from 2006 to 2022, categorized by year for the arthroscopy and arthrotomy OPS codes. Dots represent observed number of cases.

The joinpoint analysis of surgical procedures utilizing arthroscopy or arthrotomy showed initial increases for both techniques (Figure 3). The number of arthroscopies exhibited a significant increase represented by a distinct slope peaking around 2013, with approximately 9000 procedures. However, in the following years, there is a noticeable decline in the number of arthroscopies, displayed by a downward slope until 2022 (Figure 3). Also, arthrotomies showed a steady increase from 2006, reaching a peak after 2014, with approximately 1200 procedures.

**Figure 4:**
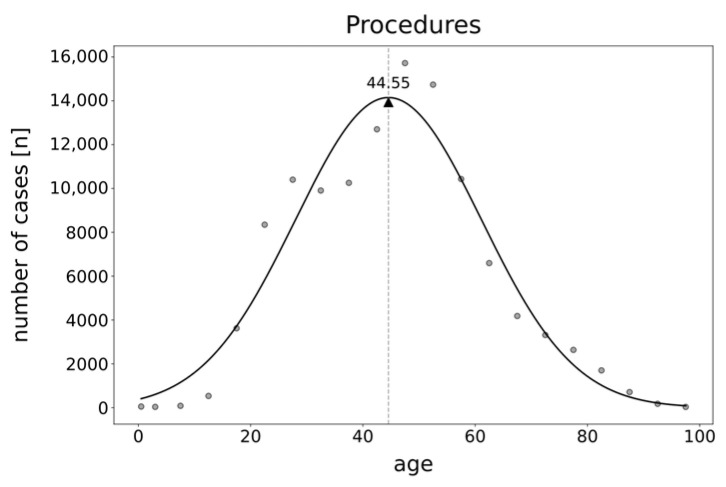
Gaussian distribution analysis of cartilage repair techniques in the hip from 2006 to 2022, categorized by patient age. Triangle represents calculated age maximum. Dots represent observed number of cases.

The age distribution for all procedures showed a monomodal pattern with patients averaging 45 years old (calculated age maximum: 44.55 years) (Figure 4).

## 4. Discussion

The present study provides comprehensive data regarding the demographic patterns and trends in cartilage repair techniques for chondral defects in the hip in Germany over a 17-year period. We demonstrated distinct differences in frequency, age and gender distribution of various surgical procedures.

Within the study period, there was a distinct increase in arthroscopic procedures as compared to arthrotomy, reflecting a continuing trend in orthopedics towards minimally invasive surgery. As hip arthroscopy evolved, a shift from pathology resection to anatomic repair and reconstructive procedures occurred [18]. Thus, arthroscopy has become the most commonly utilized operative method for cartilage defects in the hip, especially for the treatment with chondroplasty or microfracture. In a controlled retrospective study, the effectiveness of arthroscopic debridement versus arthroscopic autologous chondrocyte transplantation (ACT) for the treatment of chondral lesions was compared. The Final HHS was significantly higher in the ACT group [14].

Within the present study, men were treated more frequently than women for any of the included procedures. Indeed, younger males often exhibit reduced femoral head–neck offset, a non-spherical femoral head, or femoral retrotorsion. These conditions seem to result in a higher rate of symptomatic FAI compared to females [19].

The age distribution data exhibited distinct patterns based on the type of procedure, whereby debridement procedures showed a peak for middle-aged people. The question of up to what age hip arthroscopy is medically sensible and beneficial has not yet reached consensus in the literature. However, most studies have revealed only short-term outcomes for patients older than 50 years [19]. The peak for refixation procedures in older patients might be related to the treatment of osteochondral fractures. Interestingly, for regenerative techniques, a peak was observed for younger patients averaging 28 years old, indicating their indications in early-stage cartilage lesions. Indeed, a prospective study analyzing data from the Danish Hip Arthroscopy Registry (DHAR) for FAI procedures demonstrated that patients equal to or older than 25 years had poorer outcomes measured clinically by patient-reported outcome measures (PROMs) when compared with the age group younger than 25 years [20].

In a retrospective single-center level IV case series of 25 patients with 2–4 cm^2^ full-thickness chondral lesions undergoing hip arthroscopy for FAI, the AMIC procedure showed good clinical and radiological outcomes in a 2-year follow-up [21]. Another retrospective study compared microfracture and AMIC in an 8-year follow-up of patients with hip chondral lesions associated with FAI [22]. AMIC showed significantly better outcomes as measured by the modified Harris hip score (mHHS), which were retained after 8 years, whereas in the microfracture group, the improvement deteriorated after 1 year. However, with an inclusion criterium of age between 18 and 55 years, patients were not matched and the collagen matrix reimbursement by the payer determined the treatment patients received [22]. Moreover, a prospective study investigating arthroscopic treatment of FAI caused full-thickness cartilage defects in the hip with injectable MACI in 29 patients with a mean age of 30 years found a significantly improvement in patient-related outcome at an average follow-up of 19 months [23]. These findings support the outcome of the present study that regenerative procedures are primarily conducted in younger patients as sustained clinical outcomes are expectable. In another retrospective single-center study comparing the clinical outcomes of arthroscopic MACI with AMIC for acetabular chondral defects resulting from FAI, both procedures showed significant mHHS improvements, which remained steady by the 5-year follow up without significant differences between the groups [24]. These findings are particularly interesting because AMIC as a one-stage procedure is potentially associated with a lower rate of perioperative complications and lower costs. When comparing different joint-preserving cartilage repair techniques in the hip, the rates of conversion to total hip arthroplasty (THA) and revision arthroscopy can be useful. Regarding hip arthroscopy for FAI, a systematic review found that compared with microfracture, AMIC resulted in significantly lower rates of conversion to THA and of revision arthroscopy. In addition, patients with Outerbridge grade III and IV lesions showed significantly less improvement in PROMs and had a significantly higher rate of conversion to THA compared to those with Outerbridge grade I and II lesions [25].

The joinpoint regression analysis exhibited trends in the number of cases for different procedures over time. The strong initial increase followed by a decrease in both debridement and refixation procedures around 2013 may reflect the establishment of hip arthroscopy as a progressively popular technique for the treatment of pathological conditions. Indeed, the description of FAI as a mechanism for the development of early OA for most nondysplastic hips is relatively contemporary, as Ganz et al. proposed it in 2003 [26]. Thus, the indications and numbers of cartilage repair procedures have been extended vastly.

As a limitation of the present study, the data provided by the Federal Statistical Office of Germany did not indicate second-time procedures, and thus, reports on complications requiring revision procedures are not displayed. Apart from that, the study included only in-hospital data of fully inpatient treatment; thus, outpatient procedures are not shown. Presumably, the subsequent decline in procedures after 2013 may be explained by the elaboration of indications for these procedures. Also, as the surgical procedures and postoperative care become more standardized, some procedures for selected indications may be implemented in outpatient settings. For regenerative techniques, the distinct peak and following decline around 2017 might reflect an initial excitement and subsequently more selective indication for these procedures.

Still, a systematic review on the cost-effectiveness of hip arthroscopy for the treatment of FAI syndrome and labral tears suggested a higher initial cost but greater improvement in quality-adjusted life-year for hip arthroscopy compared to nonoperative treatment [27]. Indeed, a multicenter randomized controlled trial comparing hip arthroscopy versus best conservative care in FAI treatment demonstrated a significantly higher hip related quality of life 12 months after randomization measured by the patient-reported International Hip Outcome Tool (iHOT-33) [28]. Thus, joint-preserving cartilage repair techniques are efficient treatment options for chondral lesions, potentially postponing THA in the selected patients.

## 5. Conclusions

Overall, the findings that cartilage repair techniques in the hip were mostly performed using arthroscopy rather than arthrotomy taken together with the advancing application of regenerative procedures reflect a shift towards minimally invasive and scaffold-based treatments. The data underline the evolving field of cartilage repair techniques for chondral defects in the hip. Further research should focus on prospective comparative studies including PROMs, the quality of the repair tissue, and cost–benefit analyses to guide clinical decision-making.

## Figures and Tables

**Table 1:** OPS codes according to the German procedure classification (OPS classification system) for included procedures.

Procedure	Arthroscopy	Arthrotomy
Debridement		
Chondroplasty	5-812.eg	5-801.gg
Excision of diseased cartilage	5-812.0 g	5-801.0 g
Microfracture, subchondral drilling	5-812.fg	5-801.hg
Refixation	5-812.3 g	5-801.[3 g, 4 g]
Regeneration		
Matrix-induced autologous chondrocyte transplantation (MACI)	5-812.hg	5-801.kg
Autogenous matrix-induced chondrogenesis (AMIC)	5-812.[gg, mg]	5-801.[ng, pg]
In vitro tissue culture transplantation	5-812.ag	5-801.cg
Cartilage transplantation	5-812.9 g	5-801.bg

**Table 2:** Allocation of the surgical interventions and sexes of analyzed cases. Summary of the overall number of surgical treatments (Σ) conducted from 2006 to 2022, shown by type of procedure (arthroscopy and arthrotomy) and patient sex (male and female).

	Σ	Male	Female
Arthroscopy	98,916	54,392	44,524
Arthrotomy	17,263	9379	7884

**Table 3:** Joint-preserving surgical interventions on hip articular cartilage between 2006 and 2022, categorized by arthroscopy or arthrotomy and sex.

Procedure	Σ	Arthroscopy		Arthrotomy	
		Female	Male	Female	Male
Debridement					
Chondroplasty, excision of diseased cartilage	61,423	24,940	29,565	3164	3754
Microfracture, subchondral drilling	22,058	8225	10,850	962	2021
Σ	83,481	33,165	40,415	4126	5775
Refixation					
Σ	7978	911	1033	3418	2616
Regeneration					
Matrix-induced autologous chondrocyte transplantation (MACI)	740	51	145	97	447
Autogenous matrix-induced chondrogenesis (AMIC)	1171	371	475	117	208
In vitro tissue culture transplantation, cartilage transplantation	277	38	88	57	94
Σ	2188	460	708	271	749

## Data Availability

The original data shown in the publication are openly available in GitHub at https://github.com/ioannis-stratos/Hip, accessed on 30 July 2024.
